# Heparan Sulfate Proteoglycans and Viral Attachment: True Receptors or Adaptation Bias?

**DOI:** 10.3390/v11070596

**Published:** 2019-07-01

**Authors:** Valeria Cagno, Eirini D. Tseligka, Samuel T. Jones, Caroline Tapparel

**Affiliations:** 1 Department of Microbiology and Molecular Medicine, University of Geneva Medical School, 1205 Geneva, Switzerland; 2 School of Materials, University of Manchester, Manchester M13 9PL, UK

**Keywords:** viral attachment receptor, heparan sulfate proteoglycans, HSPG, syndecans, glypicans, viral adaptation, intra-host adaptation, tropism, broad-spectrum antivirals, viral binding

## Abstract

Heparan sulfate proteoglycans (HSPG) are composed of unbranched, negatively charged heparan sulfate (HS) polysaccharides attached to a variety of cell surface or extracellular matrix proteins. Widely expressed, they mediate many biological activities, including angiogenesis, blood coagulation, developmental processes, and cell homeostasis. HSPG are highly sulfated and broadly used by a range of pathogens, especially viruses, to attach to the cell surface. In this review, we summarize the current knowledge on HSPG–virus interactions and distinguish viruses with established HS binding, viruses that bind HS only after intra-host or cell culture adaptation, and finally, viruses whose dependence on HS for infection is debated. We also provide an overview of the antiviral compounds designed to interfere with HS binding. Many questions remain about the true importance of these receptors in vivo, knowledge that is critical for the design of future antiviral therapies.

## 1. Structure and Synthesis of Heparan Sulfate Proteoglycans

**Figure 1:**
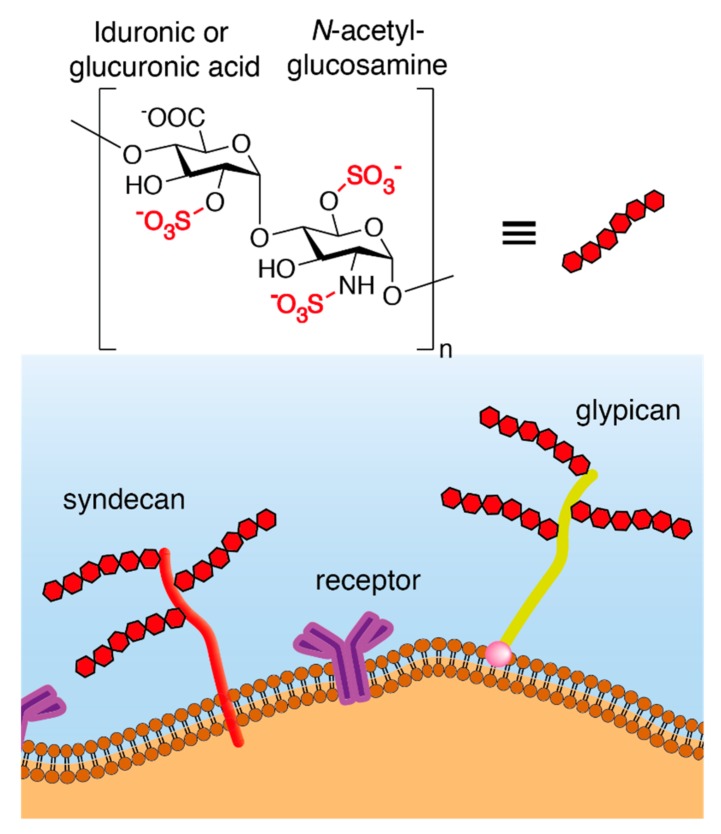
Schematic structure of syndecans and glypicans, the two HSPG chiefly involved in virus infection. HSPG typically consist of a core protein and GAG chains. The core protein of syndecans is composed of an extracellular domain, a single transmembrane domain, and a short cytoplasmic domain that interacts with the cytoskeleton. Glypicans are GPI-anchored HSPG. The GAG chain is composed of unbranched anionic polysaccharides composed of repeating disaccharide units formed by sulfated uronic acid and hexosamine residues.

Heparan Sulfate Proteoglycans (HSPG) are composed of a core protein covalently linked to glycosaminoglycan (GAG) chains formed by unbranched sulfated anionic polysaccharides, known as heparan sulfates (HS) (Figure 1). In addition to HS, other GAGs include chondroitin sulfates, dermatan sulfates, keratan sulfates, and hyaluronic acids.

The majority of HSPG are anchored in the plasma membrane of almost all eukaryotic cells, except for perlecan, agrin, and collagen XVII, which are present in the extracellular matrix (ECM) [1].

Syndecans and glypicans (Figure 1) are the two main classes of membrane-anchored HSPG. Syndecans are encoded by 4 different genes and represent the most abundant transmembrane HSPG. Their core protein is composed of an extracellular domain, a single transmembrane domain, and a short cytoplasmic domain that interacts with the cell cytoskeleton. Glypicans are encoded by 6 different genes and are anchored in the cell membrane via glycosylphosphatidylinositol (GPI).

**Figure 2:**
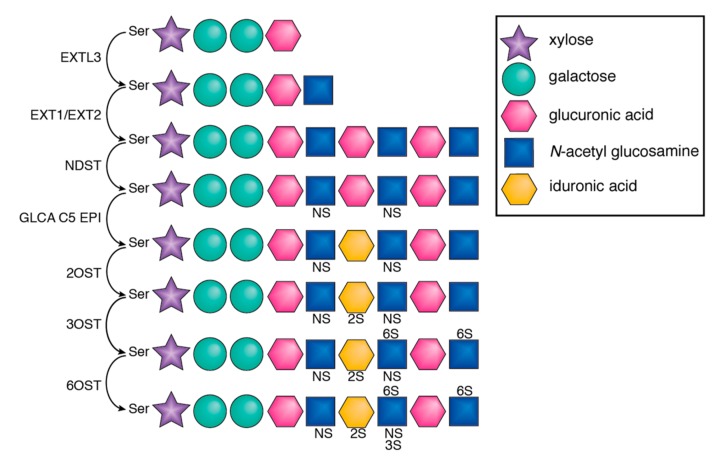
HS synthesis pathway. The glycans are attached to the protein core through a serine linker. After the addition of different sugars, *O*- and *N*-sulfotransferases further modify the side chain conferring the negative charges.

The synthesis of HSPG is initiated through the attachment of the first tetrasaccharide (i.e., xylose, galactose, galactose, glucuronic acid) to a serine residue (Ser) of the core protein (Figure 2) and the subsequent addition of *N*-acetyl glucosamine (GlcNAc) and glucuronic acid (GlcA). Five different glycotransferases, termed exostosins (EXT1, EXT2, EXTL1, EXTL2, EXTL3), mediate these attachments. The *N*-acetyl group of GlcNAc is then replaced by a sulfate group by 4 *N*-deacetylase/*N*-sulfotransferases (NDST1, NDST2, NDST3, NDST4). The subsequent step involves the epimerization of GlcA to iduronic acid (IduA) by glucuronyl C5-epimerase (GCLA C5 EPI). Finally, *O*-sulfotransferases (OST), i.e., 2-OST, 6-OST, and 3-OST, further modify the HSPG [2]. As a result, the organs of the human body contain different isoforms of HSPG with various saccharide compositions and sulfation patterns [3,4].

Several enzymes are involved in the degradation of HSPG, in particular heparanase-1, which is involved in physiological and pathological roles [5]. Similar enzymes are expressed also by bacteria and used as a tool to modify HSPG on the cell surface to investigate involvement in viral infection.

## 2. Physiological Functions of Heparan Sulfate Proteoglycans

**Figure 3:**
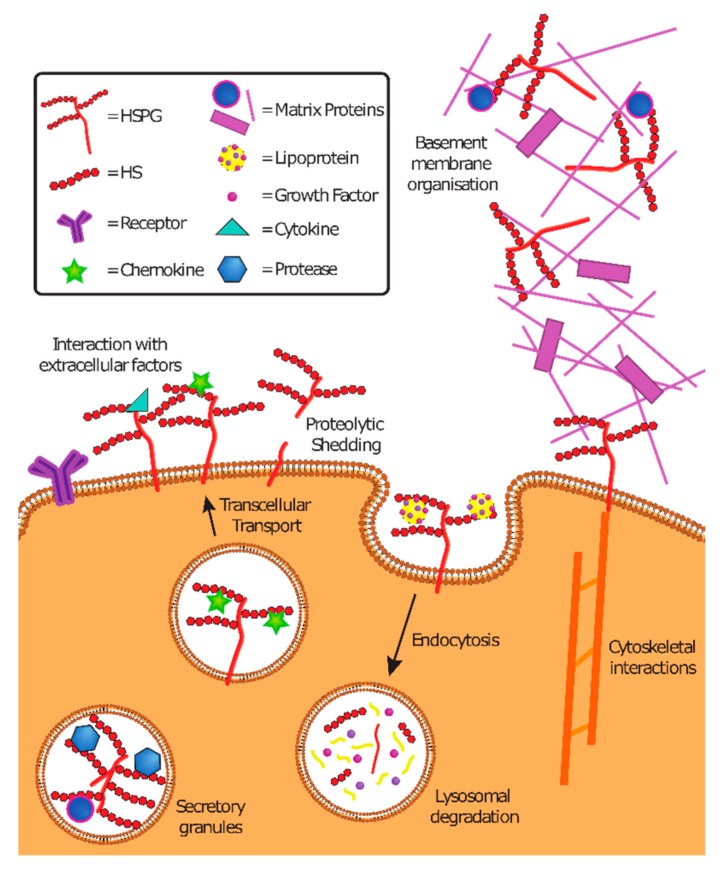
Functions of HSPG. HSPG in the ECM contribute to basement membrane organization. HSPG expressed on the cells mediate interactions with extracellular factors, play a role in endocytosis and lysosomal degradation and transcellular transport, and can be shed in response to stress after proteolytic cleavage. Adapted with permission from [7].

HSPG are conserved among vertebrates and invertebrates and have multiple functions (Figure 3). As components of the ECM, they contribute to basal membrane organization and mediate cell adhesion and motility. As part of secretory vesicles (serglycin in particular), they ensure the correct functioning of the packed content (i.e., proteases or matrix proteins). At the cell surface, HSPG bind to cytokines, chemokines, growth factors, and morphogens, preventing their degradation, thus creating temporary storage sites or gradients of morphogens important in development. Expressed at the cell surface, they also serve as endocytosis receptors, and thereby regulate the lysosomal degradation of extracellular molecules and provide nutrients to cells. Moreover, they are involved in the endocytosis of cellular receptors. They mediate the transcellular transport of chemokines across endothelial cells. They also serve as co-receptors of fibroblast growth factor (FGF) and FGF receptor in both *cis* (if expressed on the same cell) or *trans* (if expressed on different cells). They mediate intracellular signaling or intracellular stress through proteolytic shedding of syndecans. They have an important role in development and in maintaining stem cell niches.

More detailed descriptions of HSPG physiological, pathological, and morphological functions are beyond the aim of this review and can be found in [1,4,6,7].

## 3. Heparan Sulfate Proteoglycans As Viral Receptors

In mammals, HSPG are ubiquitously expressed by most cell types. Due to the heavily sulfated GAG chains, they present a global negative charge that can interact electrostatically with the basic residues of viral surface glycoproteins or viral capsid proteins of non-enveloped viruses. Viruses exploit these weak interactions to increase their concentration at the cell surface and augment their chances of binding a more specific entry receptor [8]. In rare cases, HSPG serve directly as entry receptors, as described for herpes simplex virus (HSV)-1 [9]. HSPG-dependent viruses can be grouped in distinct categories, and Table 1 lists examples of each category.

## 4. Viruses for Which Heparan Sulfate Proteoglycans Dependence Has Been Proven for Laboratory and Natural Isolates

Here we describe the HSPG dependency of HSV, human papillomavirus (HPV), and dengue virus (DENV). We selected these viruses because numerous publications support the interaction, including experiments with clinical isolates and/or in vivo data.

### 4.1. Herpes Simplex Virus

HSV belongs to the *Herpesviridae* family that includes large, enveloped, double-stranded DNA (dsDNA) viruses whose manifestations range from asymptomatic infections or mild mucocutaneous lesions on the lips, cornea, genitals, or skin to more severe, even life-threatening infections, such as encephalitis and disseminated infections in neonates or immunocompromised hosts. HSV-1 causes mainly herpes labialis and is transmitted by intimate oral contact, while HSV-2 causes herpes genitalis and is transmitted by sexual intercourse. Following primary infection of the genital or oral mucosa, HSV establishes latent infections in neurons of the sensory ganglia, from which it may reactivate and cause recurrent lesions at the site of primary infection [82]. Currently, no vaccine is available, but active antivirals, such as acyclovir, which interfere with viral DNA synthesis, exist [83].

Both HSV-1 and HSV-2 use HSPG to attach to the cell surface. The absence of HSPG expression does not totally abolish the infection rate, but decreases it significantly, as the virus rarely binds directly its entry receptor [84]. Two viral surface glycoproteins, gB and gC, first interact with HSPG. In particular, HSV-1 interacts with the HSPG-rich filopodia-like structures on primary conjunctival epithelial cells [85]. In neurons, this interaction enables the virus to move along protrusions, a process referred to as “viral surfing,” and reach the cellular body, where its entry receptors are expressed. A specific interaction between gD and different receptors [nectin-1 or nectin-2 (members of the immunoglobulin family), HVEA (herpesvirus entry mediator, a member of the tumor necrosis factor [TNF] proteins) [9,84,86] mediates the entry. The receptors used differ depending on the cell type. Interestingly, 3O-HS (3-*O*-sulfated HS, a type of HSPG) is sufficient to mediate HSV-1 attachment and fusion in primary corneal fibroblasts, where nectin-1 and HVEA are not expressed [87]. Moreover, a soluble form of 3O-HS can mediate HSV-1 infection in CHO-K1, a cell line to which the virus can attach but not enter [88]. After binding with the entry receptors, gD undergoes a conformational change that allows viral fusion. The surface glycoproteins gH and gL also play a fundamental role in this process, forming a fusion complex, together with gD and gB, necessary for release of the tegument proteins and viral DNA into the host cell [89]. Interestingly, recent studies have highlighted that HSV induces heparanase overexpression in infected cells to facilitate detachment and spreading. This mechanism prevents virus trapping at the cell surface through receptor binding, as does the cleavage of sialic acid by influenza neuraminidase [90,91].

Several sulfated molecules (i.e., heparin, carrageenan, dendrimers, nanoparticles, and others) inhibit HSV infection, further supporting the role of HS in viral attachment [27,92,93,94] (Table 2).

### 4.2. Human Papillomavirus

Human papillomavirus (HPV) is a small, non-enveloped dsDNA virus in the *Papillomaviridae* family. It infects the epithelial cells of the skin and the anogenital or oropharyngeal mucosa, causing benign or malignant neoplastic lesions. HPVs are the most common sexually transmitted viruses and are subdivided into low-risk types, which can cause low-grade cervical lesions and genital warts, and high-risk types, which cause cervix carcinoma, as well as anus, vulva, vagina, penis, and oropharynx cancers. Vaccines are available but there is no approved antiviral.

Several types of HPV, such as HPV-16, HPV-18, HPV-31, HPV-45, HPV-6, and also bovine papillomaviruses, are dependent on HSPG both in vitro and in vivo. It is believed that all papillomaviruses depend on HSPG for their initial attachment; however, due to its oncogenic potential and prevalence, HPV-16 is the serotype whose entry is most studied [20,105,112,115].

HPV starts its infectious cycle from the basal membrane of the vaginal mucosa, exploiting abrasions or lesions in the epithelium. The first contact is mediated mainly by HSPG expressed on the cellular surface of basal keratinocytes or on the ECM. A role for laminin-332 (formerly termed laminin-5) in the attachment has also been proposed [121,122,123]. Syndecan-1 plays a major role in this initial attachment, due to its expression on epithelial cells and its overproduction during wound healing [124]. The dependence on HSPG has been demonstrated not only in vitro, but also in vivo, in mice pretreated topically with heparinase III, or treated at the time of infection with heparin and then infected with HPV-16, HPV-5, and HPV-31. In presence of these treatments, a significant decrease in infection was observed [125]. The critical residues for the interaction between HSPG and HPV have been mapped as Lys278 and Lys361 on the exposed portion of the capsid protein L1 [126].

After contact with HSPG, the HPV capsid undergoes conformational changes assisted by extracellular cyclophilin B [127] and cleavage of the L2 capsid protein by furin [128]. This leads to the loss of affinity for HSPG and binding to different secondary receptors. Identification of the internalization receptor is ongoing, but α6 integrins, epidermal growth factor receptor (EGFR) and the tetraspanins family may be involved [129]. The entry kinetics of HPV appear to be asynchronous and slower than for the majority of other viruses, but the cause is still not fully understood [20,111,130]. Some research suggests that it may be linked to the cell cycle phase or the engagement of multiple receptors [129]. Subsequently, the virus is internalized through endocytosis, but there are contradictory reports on different HPV types and cells. However, entry of HPV-16 -18 and -31 pseudovirions in epithelial cells is independent of clathrin and caveolae [129,131] suggesting a common endocytic pathway for all HPV.

Several HSPG-mimicking or -interacting molecules such as small molecules, dendrimers, carrageenan, heparin, and nanoparticles [92,105,112,113,115,117] have proven to be effective for preventing HPV infection (Table 2). A carrageenan-based gel was tested in the Carraguard phase IIB clinical trial, where the treated arm showed enhanced protection when compared with the placebo arm. However, despite the promising effectiveness of the microbicide against HPV in the phase IIB clinical trial, no phase III clinical trial was performed [132]. This may be linked to the failure of such trials with similar compounds against HIV (Table 2).

### 4.3. Dengue Virus

Dengue virus (DENV) is an enveloped, positive-sense single-stranded RNA (ssRNA+) virus of the *Flaviviridae* family, which is composed of 4 distinct serotypes (DENV1, DENV2, DENV3, DENV4). Billions of people live in areas at risk of dengue transmission, and approximately 390 million DENV infections are reported yearly, leading to 25,000 deaths [133]. The virus is mainly transmitted by *Aedes* mosquito bites. Infected individuals can be asymptomatic or display dengue fever, a self-limiting disease. However, especially after secondary infections by a different DENV serotype, the virus can cause dengue hemorrhagic fever (DHF) and dengue shock syndrome (DSS), both of which can lead to death.

The first demonstration of DENV binding to HSPG was reported in 1997, where highly sulfated liver-derived HS, as well as heparin, were shown to inhibit DENV2. Moreover, the alteration of GAG expression or sulfation on cells reduced DENV infectivity, as demonstrated through treatment of Vero cells with heparinase I and III or sodium chlorate (a sulfation inhibitor) and through infection in psg-D677 and psg-A745 CHO cell lines, which lack HSPG synthesis [134]. The HSPG dependence has also been demonstrated by competition experiments with numerous sulfated molecules (see Table 2) [92,101,102,134]. The interaction site with HSPG has been mapped subsequently to domain III of the dengue envelope (E) protein, on an external loop region rich in surface basic residues [135]. Over time, numerous papers have been published on the interaction between the E protein and HSPG in human endothelial, liver, and animal cell lines [136,137,138]. It is unclear if there is also an interaction with HSPG in insect cell lines (C6/36) [139,140]. Moreover, to exclude any effect linked to cell adaptation, DENV pseudoparticles produced with the sequences of E and pre-matrix (prM) proteins of viruses never passaged in cells have been shown to bind HSPG and heparin in a manner comparable to that of viruses extensively passaged in cell lines [17].

In human cell lines, HSPG are well proven to be the first attachment receptors, followed by more specific interactions with one of the known entry receptors such as dendritic cell–specific intercellular adhesion molecule-3-grabbing non-integrin (DC-SIGN), the mannose receptor (MR) for macrophages, and other proteinaceous receptors [141]. The variety of receptors demonstrated to be involved in DENV entry may be related to the wide variety of tissues infected (liver, lymph node, spleen, bone marrow) [141].

## 5. Viruses That Show Attachment to Heparan Sulfate Proteoglycans After Cell Adaptation

Some viruses do not use HSPG as receptors in vivo but become HSPG-dependent after repeated passage in cell culture. Many cell lines express HSPG abundantly. Given their error-prone replication machinery, viruses can rapidly mutate. A possible outcome is an increase in the number of basic residues on their surface protein, leading to acquisition of the ability to use negatively charged GAG chains as attachment receptors. This results in improved viral fitness and the out-competing of HSPG-independent variants.

### Rhinoviruses and Other Picornaviruses

Rhinoviruses (RVs) are small ssRNA+ viruses in the *Enterovirus* genus in the *Picornaviridae* family. They are the main causes of acute viral infections worldwide. In immunocompetent individuals, RVs typically induce the common cold with nasal congestion and rhinorrhea, coughing, sneezing, sore throat and malaise, and spontaneous resolution within 1–2 weeks [142]. However, RVs can also cause a wide range of complicated illnesses, such as exacerbation of asthma [143,144] and of chronic obstructive pulmonary disease [145,146], pneumonia, and bronchiolitis [147], as well as chronic infections in immunocompromised hosts, with fatal outcome in some cases [148,149]. This group of viruses consists of numerous types that are organized into 3 species: RV-A (80 types), RV-B (32 types), and RV-C (56 types). Most RV-As and all RV-Bs bind intercellular adhesion molecule 1 (ICAM1) [150] for cell entry and belong to the major group RVs, while 11 RV-As use the low-density lipoprotein receptor family (LDL-R) and belong to the minor group RVs [151]. Some major group RVs (RV-8, -54, -89) can also use HSPG as an additional receptor [55,60,65] either directly or after multiple passages in cells lacking ICAM1. For the HSPG-dependent variants, increased susceptibility to acidic pH and elevated temperatures suggest that the greater instability might compensate for the absence of the uncoating activity of ICAM1 and the loss of virulence in vivo [60]. The third RV species, RV-C, uses cadherin-related family member 3 (CDHR3) to infect the cell [152]. CDHR3 is specifically expressed on ciliated cells of airway epithelia, explaining why RV-Cs do not grow in standard cells [153]. However, exogenous expression of CDHR3 in HeLa cells renders the cells permissive to RV-C. Serial passages of RV-C15 in these cells leads to increased viral binding, viral yields, and stronger cytopathic effects. A mutation in the external surface of the VP1 capsid protein, T125K, was identified in the majority of the adapted viral population and was demonstrated to confer enhanced HS-mediated cell binding [49]. The adapted virus was also able to replicate in HeLa cells lacking CDHR3, although to a lower extent, as well as in primary differentiated bronchial cells.

In addition to rhinoviruses, members of the enterovirus (EV)-B species, such as Coxsackie virus B3 (CV-B3) [154], culture-adapted echoviruses, low-passage clinical echovirus 6 isolates, and echovirus 5 bind HSPG [35]. The natural receptors of CV-B3 are decay-accelerating factor (DAF) and the coxsackie adenovirus receptor (CAR), but it is possible to select, through cell adaptation, a HS-binding variant. This virus is associated with mutations in VP1 and a change of cellular tropism with lytic infection in a wider spectrum of cell lines as opposed to the parental strain. Similarly to CV-B3, foot-and-mouth disease virus (FMDV), a member of the *Aphthovirus* genus in the *Picornaviridae* family, also adapts easily to HS binding during tissue culture through the selection of positive charge–containing amino acids within the capsid protein. In conclusion, despite the majority of *Picornaviridae* family members being reported to bind other receptors, some, especially after cell culture adaptation, are able to bind HSPG.

Of note, as these viruses can become HSPG-dependent in cell lines, similar adaptations may occur during human infections to promote replication in HSPG-enriched tissues. The next paragraph highlights some examples.

## 6. Viruses with Acquired Dependence on Heparan Sulfate Proteoglycans after Human Intra-Host Adaptation

Each infected host presents a different environment that may favor the selection of new and fitter viral variants. To follow this intra-host adaptation, viral sequencing must be performed at different times and in different sites during the disease course. In the case of enterovirus 71 (EV-A71) and John Cunningham polyomavirus (JCV), the original clinical strains did not use HSPG as primary receptors, but mutant viruses binding HSPG were isolated from the central nervous system of patients.

### 6.1. Enterovirus 71

EV-A71 is a non-enveloped ssRNA+ virus in the *Picornaviridae* family. It is typically transmitted via the fecal–oral route, but in countries with high hygiene levels, transmission also occurs via the respiratory route [155]. EV-A71 is one of the major causative agents of the mild and self-limiting hand, foot, and mouth disease (HFMD) outbreaks in the Asia-Pacific region [6,156,157,158]. In rare cases, particularly in immunocompromised patients or children below 6 years old, the virus can disseminate to the central nervous system, leading to severe and fatal neurological complications [159,160]. No effective antiviral treatment is currently available for EV-A71 infections. However, the Chinese Food and Drug Administration (FDA) has approved two inactivated vaccines against the C4 genogroup, which is the most prevalent genogroup in these areas [161].

EV-A71 can infect cells via two entry receptors that trigger viral uncoating and RNA release in the cytoplasm: SCARB2 (scavenger receptor class B member 2), a major transmembrane lysosomal protein [162], and PSGL-1 (P-selectin glycoprotein ligand-1), which is primarily expressed on leukocytes and interacts with specific EV-A71 strains [163,164]. In addition, EV-A71 can use a variety of different attachment receptors such as HSPG [68], sialic acids [165], nucleolin [152,166], vimentin [140,167], and annexin II [168]. These receptors enhance viral infectivity and may contribute to viral dissemination and neurotropism.

Viral genome analysis of clinical specimens isolated directly from an immunocompromised patient with disseminated EV-A71 infection has revealed intra-host adaptation [22]. A single mutation in the VP1 capsid protein, i.e., a substitution of the neutral leucine with a positively charged arginine (VP1_L97R_), was present in the blood and the cerebrospinal fluid (CSF) and in the gastrointestinal tract, while it was absent from respiratory tract specimens of the immunocompromised patient. As opposed to the VP1_97L_ variant, VP1_97R_ showed HSPG-binding ability [23] and was inhibited by highly sulfated carrageenans in Vero cells and neural cell cultures, underlining the fact that HS analogs can be promising compounds for preventing EV-A71 replication and dissemination. These data sets suggest EV-A71 adaptation in vivo towards a HS-dependent variant that promotes viral dissemination and neurotropism in an immunosuppressed patient [23].

### 6.2. Polyomaviruses

Polyomaviruses belong to the family of *Polyomaviridae* and are non-enveloped dsDNA viruses. These viruses persistently infect the majority of the population. The primary infection occurs in early childhood through person-to-person contact or through contaminated surfaces. However, healthy individuals do not show any signs of disease, while in immunocompromised patients, the viruses can reactivate and cause pathologies. Currently, no vaccines or antivirals are available for polyomaviruses [169]. The majority of these viruses can hemagglutinate erythrocytes due to their binding to sialic acid, which is exposed on gangliosides. The only member of the family reported to bind HSPG naturally is Merkel cell polyomavirus, a virus associated with a lethal skin cancer in the elderly and in the immunocompromised [170]. The virus is dependent on HSPG in the initial attachment phase, as demonstrated by the lack of binding to HSPG-deficient CHO cell lines and the inhibition after heparinase treatment. In a subsequent step, the virus interacts with sialic acid, which mediates entry [29].

Another member of the *Polyomaviridae* is JCV. In immunocompromised individuals such as AIDS patients, JCV is responsible for progressive multifocal leukoencephalopathy (PML) leading to rapid demyelination in the central nervous system, with consequent cognitive impairment and motor dysfunctions [169]. JCV receptors on cells are sialic acid residues on the lactoseries tetrasaccharide c (LTSc pentasaccharide) and the serotonin receptor 5-HT 2A R [171,172]. However, virus isolated from the urine of healthy subjects differs from that isolated from patients with PML in the form of mutations in the major capsid protein VP1 [13,173]. A recent study showed that these mutants have increased HSPG binding activity, as demonstrated by decreased ability to bind cells after heparinase treatment and increased heparin inhibition when compared to the parental non-mutated strain. These mutations and differences in binding can confer the ability to infect neural cells, which are known to express high levels of glypicans and syndecans [13].

## 7. Viruses with Controversial Data on Heparan Sulfate Proteoglycans Dependence

The actual HS dependence of some viruses is still debated due to their relatively new emergence or re-emergence, such as Zika virus (ZIKV), or to the probable lack of HSPG on their natural sites of infection, such as respiratory syncytial virus (RSV). Of note, as viral receptors are mostly investigated in cell lines with laboratory adapted viruses, some of these controversies may also be the result of in vitro adaptations.

### 7.1. Respiratory Syncytial Virus

Human RSVs are enveloped ssRNA- of the *Orthopneumovirus* genus in the *Pneumoviridae* family. They are the primary cause of bronchiolitis and pneumonia in children under 5 years of age and are also linked to respiratory complications in the immunocompromised and the elderly. It has been estimated that, in 2015, RSV caused (globally) acute lower respiratory infections in 33.1 million children aged less than 5 years, with 3.2 million hospital admissions and 59,600 deaths [174]. No vaccine is currently available, and the only approved specific treatment is palivizumab, a monoclonal antibody directed against RSV fusion (F) protein. This immunoglobulin is used only as prophylaxis and is limited to pre-term, low-weight, immunocompromised infants [175,176].

RSV binding to cells involves an initial interaction between the basic amino acids present on the viral envelope proteins G and F and the negatively charged HSPG [177,178,179]. Subsequently, the virus engages secondary receptors: nucleolin [180], ICAM1 [181], C-X3-C motif chemokine receptor 1 (CX3CR1) [182], and annexin II [183]. These interactions induce a conformational change of the F protein with exposure of its fusion peptide, promoting fusion of the viral envelope and the cell plasma membrane and release of the nucleocapsid in the cytoplasm [184].

Competition experiments with heparin and other sulfated molecules, enzymatic removal of HSPG from the cellular surface, as well as inhibition of sulfation have shown that RSV initial attachment to cells is HSPG-dependent [118,177,178] (Table 2). However, recent studies on human airway epithelial (HAE) cultures have shown that HS have limited expression on the apical side compared to the basal side [23,26], and RSV is known to infect mainly apical ciliated cells of the respiratory epithelia [14,15]. Therefore, it has been proposed that RSV can bind different receptors in cells and in HAE, where CX3CR1 is sufficient for mediating the infection [14,185].

Nonetheless, some compounds related to HSPG have also proven to be inhibitory in HAE [15,16] and in vivo [92] (Table 2). The CX3CR1 and HSPG binding sites are distinct but spatially close; therefore, HSPG mimetics may inhibit CX3CR1-mediated infection via steric hindrance.

To our knowledge, there has been no study in HAE with clinical isolates to investigate whether HSPG binding is linked to cellular adaptation or is a property of all RSV strains. In the latter case, this receptor may be used by the virus in vivo to infect cells expressing HSPG such as in the alveoli or in yet unidentified cells. Indeed, HAE are representative of upper respiratory tract airways, while HSPG, in particular syndecan-1 and syndecan-4, are abundant in the alveolar epithelia [186], and RSV complications are associated with lower respiratory infections.

Alternatively, restricted HSPG expression at the apical side of HAE may also be due to artifacts linked to HAE differentiation in vitro. Immunostaining of CX3CR1 and HSPG expression in human biopsies would aid understanding the specific role of each of these receptors in RSV infection.

Moreover, the same considerations can be extended to other members of the *Pneumoviridae* family, particularly human metapneumovirus (HMPV), which also binds HSPG, infects ciliated cells on HAE, and whose inhibition with sulfated molecules has also been shown in HAE [33,34] (Table 2).

### 7.2. Zika Virus

ZIKV belongs to the *Flaviviridae* family and is an enveloped ssRNA+ virus. It has been recently associated with birth defects in South America, due to the ability of the virus to cross the placental barrier and infect the neural system of the fetus, causing microcephaly [187]. Up to 580,000 suspected cases of ZIKV were reported to PAHO (Pan American Health Organization) from January 2015 to January 2018 only in the Americas [187]. Its entry mechanism has not been fully elucidated, although due to phylogenetic similarity, it is proposed to be similar to DENV.

In a study using a surface plasmon resonance (SPR) binding assay, Kim and colleagues showed that ZIKV E protein interacts with HS and chondroitin sulfates. The authors calculated that the binding constant (Kd) is 443 nM, while that of DENV E protein is 15 nM. They ascribe this variation to the higher number of basic residues on the DENV E protein as compared to that of ZIKV [39].

However, the results of successive studies that used the full virus instead of the purified E protein did not indicate a dependence of ZIKV on HSPG. In two studies, a negatively charged molecule proved to be active against ZIKV, but not through inhibition of HSPG binding [40,41].

Tan et al. reported that suramin was effective against ZIKV; however, the infectivity of ZIKV in cell lines was not diminished after sodium chlorate wash or heparinase I/III treatment, nor was it possible to observe any interaction between the virus and heparin-Sepharose beads. Through molecular dynamics simulation, the authors evidenced a strong interaction between suramin and ZIKV helicase, possibly explaining the inhibitory activity [40].

In a second study, heparin was poorly effective against ZIKV, and mainly through inhibition of the caspase III and apoptotic pathways, protecting the cells from cell death rather than having effects on viral adhesion to the cell [41].

An additional study showed that HSPG are present in mosquitoes and in different human anatomical sites known to be efficiently infected by ZIKV. Nevertheless, when the virus was pre-incubated with different heparin variants, not only there was no protection, but the viral titer was also increased [188].

Further research, in which a clinical isolate was serially passaged in Vero cells, identified the L307F mutation in domain III of the ZIKV E protein, with possible but not verified involvement in the attachment to cell HSPG, enhancing viral infectivity in combination with a mutation (M220V) in nonstructural protein 1 (NS1) [189].

These controversial results suggest that ZIKV, although closely related to DENV, is not dependent on HSPG, with the exclusion of the SPR study, where only the ZIKV E protein was used.

Contradictory results have also emerged from studies in which host genes necessary for viral replication were identified. ZIKV, as well as DENV, requires the expression of EXT1 and NDST1, two enzymes of the HS biosynthetic pathway [42]. However, another study showed that ZIKV binding to cells was not affected by inactivation of the *SLC35B2* (solute carrier family 35 member B2) gene, which is involved in the sulfation of HSPG. The same results were obtained for the glycosyltransferases B3GAT3 (beta-1,3-glucuronyltransferase 3) and B4GALT7 (beta-1,4-galactosyltransferase 7), while the inactivation of the 3 genes was effective in preventing DENV binding. However, ZIKV intracellular RNA in knockout cells was reduced at early time points, and subsequently increased, as compared to the wild-type cells, with an effect related to the inhibition of cell death [190].

Therefore, the pleiotropic effects of HSPG on the ZIKV cell cycle warrant further investigation.

## 8. Heparan Sulfate Proteoglycans Binding and Virulence

HSPG are highly expressed in almost all human tissues. Viruses that can bind this receptor are thus expected to have broader tropism. Indeed, some reports support an association between HSPG binding and virulence, while on the contrary, others show an inverse correlation between usage of this receptor and virulence or dissemination abilities. HSPG binding promotes HIV neurovirulence by allowing the infection of endothelial cells that do not express CD4 and facilitating the crossing of the blood–brain barrier [191]. Increased neurovirulence was also described for a natural isolate of North American eastern equine encephalitis virus compared to a mutant with impaired HSPG binding ability [43]. The same observation was made for a strain of Sindbis virus with a glutamate-to-histidine substitution in the E protein as compared to its non-mutated counterpart [192]. As discussed above, a HSPG-binding EV-A71 mutant was isolated from the blood and CSF of a patient with disseminated infection [23], and variants with increased numbers of positively charged amino acids in the VP1 capsid protein are also frequently isolated from patients with neurological complications [193]. Altogether, these data support an association between dissemination, neurovirulence, and HS dependency in humans.

However, several animal experiments show the opposite association. EV-A71 [194], FMDV [12], and yellow fever virus (YFV) [195] strains adapted to bind HSPG in cells are less prone to dissemination in animal models. In the same vein, after inoculation of HSPG-dependent CV-B3 [63] and DENV2 [196] variants, HSPG-independent revertants could be isolated from different sites. This link between HSPG dependency and inefficient dissemination extends to other flaviviruses (JEV [Japanese encephalitis virus], WNV [West Nile virus], MVE [Murray Valley encephalitis]) [69,70,71]. Finally, a recent study in a mouse model of hepatitis B virus (HBV) infection also corroborates a negative association between HSPG binding and viral spread. HBV undergoes a slow maturation process, in which the HSPG binding protein is exposed in a temperature- and time-dependent manner. Based on their observations, the authors suggested that HBV could use this process to avoid becoming trapped in the blood stream, where HSPG are highly expressed but the infection would be unproductive. With this strategy instead, the virus can reach the liver, and after binding HSPG, infect the cells via its receptor, sodium/taurocholate cotransporting polypeptide (NTCP) [197]. Nonetheless, these studies present several limitations. First, animal models are often genetically modified to support viral infections (i.e., IFN [interferon] receptor knockout or transgenic expression of the viral receptors), second, inoculated viruses are animal-adapted, and finally, the inoculation route and infectious doses used are far from natural conditions. Additional reports in more relevant models are thus necessary to aid understanding of the true impact of HSPG binding on viral pathogenesis in humans.

## 9. Conclusions

HSPG are widely distributed cellular receptors with various biological activities. These receptors are hijacked by numerous viruses to attach to host cells. This typically occurs through electrostatic interactions between the negative charges of HSPG and the basic amino acid portions of viral surface proteins. Here, we describe viruses with inherent or acquired ability to bind HSPG. A consistent amount of data supports the natural dependence of HSV, DENV, and HPV on HSPG for their attachment to the host cells. For other highly prevalent viruses such as FMDV, CV-B3, or the rhinoviruses RV-C15, RV-A8, and RV-A89, adaptation to HSPG occurs after extensive passaging in cells or in the absence of their primary receptor. Interestingly, some viruses such as EV-A71 and JCV can even gain HSPG-binding ability in vivo after intra-host adaptation. Finally, for other viruses such as RSV or ZIKV, HSPG binding remains controversial, and the literature contains contradictory reports. Despite extensive studies into HSPG–virus interactions, the true impact of this receptor on in vivo viral pathogenesis remains also poorly understood. In some cases, HSPG binding appears to promote dissemination and neurovirulence, while in other reports, it traps the virus and leads to attenuated infection. Additional investigations with clinical viral strains and in conditions as close as possible to native human infection will clarify these issues.

Due to their use by many distinct viruses, HSPG represent an ideal broad-spectrum antiviral target. However, several HSPG-mimicking compounds such as carrageenan, cellulose sulfate, and PRO 2000 have failed in phase III clinical trials, as their inhibitory effect was lost upon dilution in body fluids, probably leading to the release of infectious particles [107]. We recently successfully designed broad-spectrum, non-toxic antiviral nanoparticles mimicking HSPG and presenting an irreversible virucidal inhibition mechanism [92]. Additional efforts in this direction will aid the development of antiviral drugs that are effective not only on a large number of existing viruses but also on unpredictable emerging viruses.

## Figures and Tables

**Table 1:** Classification of viruses according to their HSPG dependence.

HSPG Dependence					
Proven on Natural Isolates	Proven on Laboratory Strains		From Cell Culture Adaptation	From Human Intra-Host Adaptation	Under Debate
**Herpes simplex virus** [9]	Cytomegalovirus [10]	Human herpes virus-8 (Kaposi sarcoma herpes virus) [11]	**Foot and mouth disease virus** [12]	**John Cunningham polyomavirus** [13]	**Respiratory syncytial virus** [14,15,16]
**Dengue virus** [17]	Pseudorabies virus [18]	**Human papillomavirus** [19,20]	Venezuelan equine encephalitis virus [21]	**Enterovirus 71** [22,23]	Parainfluenza virus 3 [24,25,26,27]
**Echovirus 5** [28]	**Merkel cell polyomavirus** [29]	Hepatitis C virus [30]	Sindbis virus [31,32]		Human metapneumovirus [14,33,34]
**Echovirus 6** [35]	**Hepatitis B virus/hepatitis Delta virus** [36,37]	Adeno-associated virus 2 [38]	Semliki forest virus [32]		**Zika virus** [39,40,41,42]
**North American eastern equine encephalitis virus** [43]	Vaccinia virus [44,45]	**Human immunodeficiency virus** [46,47,48]	**Rhinovirus C15** [49]		Adenovirus 5 [50,51,52]
	Adenovirus 2 [50,51]	Filoviruses [53,54]	**Rhinovirus 8** [55]		Coronavirus NL63 [56,57]
	Norovirus genogroup II [58]	Akabane virus [59]	**Rhinovirus 89** [60]		
	Schmallenberg virus [59]	Rift valley fever virus [61,62]	**Coxsackie virus B3** [63]		
	Rabies virus [64]	**Rhinovirus 54** [65]	**Yellow fever virus** [66]		
	Swine vesicular disease virus [67]	Enterovirus 71 [68]	**Japanese encephalitis virus** [69,70,71]		
	Theiler murine encephalomyelitis virus [72]	Coxsackie virus A9 [73]	**West Nile virus** [70]		
	Human parechovirus 1 [73]	Hendra and Nipah viruses [74]	Tick-borne encephalitis virus [75]		
	Porcine reproductive and respiratory syndrome virus [76]	Human T cell leukemia virus type 1 [77]	Coronavirus group 1 [57]		
	Porcine circovirus 2 [78]	Hepatitis E virus [79]	Coronavirus OC43 [57]		
			Chikungunya virus [80,81]		
			**Murray Valley encephalitis virus** [71]		

Viruses in bold are discussed in detail in the following sections.

**Table 2:** Compounds showing broad-spectrum activity by interfering with virus–HSPG interaction.

Virus	Molecule	Virus	Molecule
HSV-1	Heparin [27]	HSV-2	Heparin [27]
	Carrageenans [27]		Carrageenans [27]
	Cellulose sulfate [95]		Cellulose sulfate [95]
	PRO 2000 [96,97]		PRO 2000 ^a^ [96,97]
	SB105-A10 dendrimer [98]		VivaGel (SPL7013) ^a^ [99]
	Sulfated K5 derivatives [100]		SB105-A10 dendrimer [98]
	Agmatine-derived polymers [93]		Sulfated K5 derivatives [100]
	MUS:OT * nanoparticles [92]		Agmatine-derived polymers ^b,c^ [93]
	DSTP27 ** [94]		MUS:OT * nanoparticles ^b^ [92]
			DSTP 27 ** [94]
HCMV	Heparin [10]	DENV2	Heparin [101]
	SB105-A10 dendrimer [98]		Carrageenans [102]
	Sulfated K5 derivatives [103]		Sulfated K5 derivatives [101]
	Agmatine-derived polymers [104]		MUS:OT * nanoparticles [92]
	DSTP 27 ** [94]		
HIV	Heparin [27]	HPV	Heparin ^c^ [105]
	Carrageenans ^d^ [27,106,107]		Carrageenans ^c,e^ [105,108,109]
	Cellulose sulfate ^d^ [95,110,107]		Cellulose sulfate [111]
	PRO 2000 ^d^ [96,107]		SB105-A10 dendrimer [112]
	VivaGel (SPL7013) ^a^ [99]		Sulfated K5 derivatives [113]
	SB105-A10 dendrimer ^b^ [114]		Agmatine-derived polymers [115]
	Sulfated K5 derivatives [116]		MUS:OT* nanoparticles [92]
	DSTP27 ** [94]		DSTP27 ** [117]
RSV	Heparin [118]	HMPV	Heparin [119]
	SB105-A10 dendrimer ^f^ [16]		Carrageenans [33]
	Sulfated K5 derivatives ^f^ [15]		SB105-A10 dendrimer ^f^ [33]
	MUS:OT * nanoparticles ^c^ [92]		Sulfated K5 derivatives ^f^ [33]
	Agmatine-derived polymers [104]		
	DSTP27 ** [94]		
EBOV	Heparin [53]	MARV	Heparin [53]
	Carrageenans [53]		Carrageenans [53]
	SB105-A10 dendrimer [53]		SB105-A10 dendrimer [53]
VV	Heparin [45]	EV-A71	Carrageenans [23,120]

^a^ ex vivo (human cervicovaginal fluid), ^b^ vaginal tissues, ^c^ in vivo, ^d^ failed in phase III clinical trial, ^e^ phase IIB clinical trial, ^f^ respiratory tissues, ^g^ neural tissues, * mercaptoundecansulfonate:octanthiol, ** N,N’-bisheteryl derivative of dispirotripiperazine.
